# Exploring the biosynthetic pathway of lignin in *Acorus tatarinowii* Schott using *de novo* leaf and rhizome transcriptome analysis

**DOI:** 10.1042/BSR20210006

**Published:** 2021-06-11

**Authors:** Shengxiang Zhang, Liqiang Zhao, Chunmiao Shan, Yuanyuan Shi, Kelong Ma, Jiawen Wu

**Affiliations:** 1 Anhui University of Chinese Medicine and Anhui Academy of Chinese Medicine, Hefei 230038, China; 2 Key Laboratory of Xin’an Medicine, Ministry of Education, Anhui University of Chinese Medicine, Hefei 230038, China; 3 Clinical College of Integrated Traditional Chinese and Western Medicine, Anhui University of Chinese Medicine, Hefei 230012, China; 4 Synergetic Innovation Center of Anhui Authentic Chinese Medicine Quality Improvement, Hefei 230012, China

**Keywords:** Acorus tatarinowii Schott, lignin biosynthesis, peroxidase, transcriptome

## Abstract

*Acorus tatarinowii* Schott is a well-known Chinese traditional herb. Lignin is the major biologically active ingredient and exerts a broad range of pharmacological effects: it is an antitumor, antioxidant and bacteriostatic agent, and protects the cardiovascular system. In the present study, the transcriptomes of the leaf and rhizome tissues of *A. tatarinowii* Schott were obtained using the BGISEQ-500 platform. A total of 141777 unigenes were successfully assembled, of which 76714 were annotated in public databases. Further analysis of the lignin biosynthesis pathway revealed a total of 107 unigenes encoding 8 key enzymes, which were involved in this pathway. Furthermore, the expression of the key genes involved in lignin synthesis in different tissues was identified by quantitative real-time PCR. Analysis of the differentially expressed genes (DEGs) showed that most of the up-regulated unigenes were enriched in rhizome tissues. In addition, 2426 unigenes were annotated to the transcriptome factor (TF) family. Moreover, 16 TFs regulating the same key enzyme (peroxidase) were involved in the lignin synthesis pathway. The alignment of peroxidase amino acid sequences and the analysis of the structural characteristics revealed that the key peroxidase enzyme had well-conserved sequences, spatial structures, and active sites. The present study is the first to provide comprehensive genetic information on *A. tatarinowii* Schott at the transcriptional level, and will facilitate our understanding of the lignin biosynthesis pathway.

## Introduction

*Acorus tatarinowii* Schott (*A. tatarinowii*) is a well-known traditional Chinese herb that is abundant in eastern and southern Asia. *A. tatarinowii* is believed to have the potential to alleviate cognitive decline, epilepsy, and dementia [1]. The main biologically active ingredients are phenylpropanoids, lignins, sesquiterpenoids, and alkaloids [2,3]. The active ingredients of *A. tatarinowii* confer a broad range of pharmacological effects, including hypoglycemic, anti-Alzheimer’s disease (AD), antiepileptic, neuroprotective, and memory-enhancing activities [4,5].

Lignin is the major bioactive ingredient in *A. tatarinowii*. Lignin is a composite phenolic polymer, composed mainly of hydroxyphenyl (H), guaiacyl (G), and syringyl (S) units, which are formed through the oxidative polymerization of *p*-coumaryl, coniferyl, and sinapyl alcohols, respectively [6]. The main functions of lignin are to provide mechanical support for plants, water conductivity, and protection against plant pathogens [7,8]. Reduced lignin contents often affect lodging resistance [9]. In addition, lignin has a waterproofing effect on the cell wall, forms a vascular system for the transport of water and solutes, and participates in the protection of plants from pathogens [10]. The initial three steps of lignin biosynthetic pathway, catalyzed by phenylalanine ammonia-lyase (PAL), cinnamate 4-hydroxylase (C4H), and 4-coumaroyl CoA-ligase (4CL), are mandatory and provide the basis for all subsequent branches and resulting metabolites. PAL participates in the first step, catalyzing the nonoxidative deamination of phenylalanine to cinnamic acid and directing the carbon flow from the phenylalanine pathway [11]. Cinnamic acid forms *p*-cinnamic acid, which is catalyzed by C4H; *p*-cinnamic acid is then converted into *p*-coumaroyl-CoA and feruloyl-CoA by 4CL and caffeic acid 3-O-methyltransferase (COMT) [12]. Cinnamyl-CoA reductase (CCR) is responsible for catalyzing the most important metabolic reaction in the biosynthesis of lignin monomers [13]. Finally, monomeric lignin is catalytically synthesized by cinnamyl-alcohol dehydrogenase (CAD), peroxidase (POX), and ferulate-5-hydroxylase (F5H) [14,15]. Although researchers have studied *A. tatarinowii* for more than a century, the genomic information of *A. tatarinowii* remains incomplete.

RNA sequencing is a particularly effective technology for gene discovery, especially for species without a reference genome [16]. This technology has been used widely in the study of traditional medicinal plants, such as *Rehmannia glutinosa* [17], *Andrographis paniculata* [18], *Solanum elaeagnifolium* [19], and *Arisaema heterophyllum* Blume [20]. Comprehensive transcriptome information can be analyzed further to identify gene expression levels, gene sequences, biosynthetic pathway, and key enzymes, and to reveal candidate genes potentially involved in specific metabolic pathways. However, given that the biosynthesis of lignin and other secondary metabolites in *A. tatarinowii* is still largely unknown, further research on this species has been hindered. In the present study, we characterized the transcriptomes of the leaf tissue and rhizome tissue of *A. tatarinowii* and identified the unigenes encoding key enzymes in the lignin biosynthesis pathway. The transcriptome data for *A. tatarinowii* reported in the present study will provide an important resource for the study of lignin biosynthesis and other metabolic pathways. Furthermore, this database will provide important clues to explore the biological characteristics of other plants closely related to *A. tatarinowii*.

## Results

### RNA-Seq and assembly

In total, 124084 raw reads were obtained from the rhizome tissue and 76197 were obtained from the leaf tissue. The raw reads were generated using the BGISEQ-500 sequencing platform. The Q30 of each plant tissue was greater than 86.82%, and the GC content of each plant tissue was approximately 42.11%. In total, 141777 unigenes were assembled and clustered from high-quality reads, thorough quality control, and filtering. Among these unigenes, 51352 (36.22%) were longer than 1000 nt and 85212 (53.69%) were longer than 500 nt. The average length of the unigenes was 1108 nt and the N50 was 2062 nt. The distribution of the lengths of the unigenes is presented in Supplementary Figure S1. The quality of transcriptome sequencing was tested using a single copy orthologous database (BUSCO), and 96% of unigenes had exact matches in the BUSCO database (Supplementary Figure S2). The complete transcriptome data obtained in this sequencing have been deposited in the NCBI SRA database (accession number: SRA226668).

### Functional annotation of unigenes

**Figure 1:**
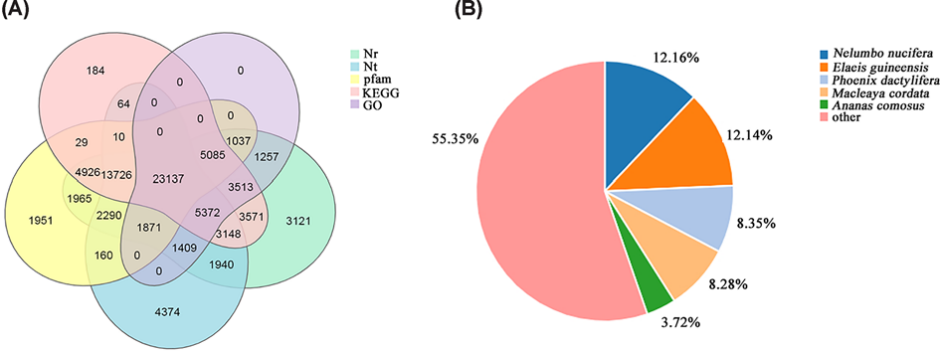
Unigene functional annotation (**A**) Venn diagram of annotated unigenes in five different databases. (**B**) Species distribution of the annotations in the Nr database for *Acorus tatarinowii*.

The 141777 identified unigenes were annotated using seven NCBI publicly available databases; the annotations were distributed as follows: Nr, 77368 unigenes (54.57%); Nt, 57501 unigenes (40.56%); Swissprot, 58557 unigenes (41.30%); KEGG, 62765 unigenes (44.27%); GO, 42681 unigenes (30.10%); KOG, 62928 unigenes (44.39%), and Pfam, 56187 unigenes (39.63%) (Table 1). A totaol of 76714 (68.22%) unigenes were annotated in at least one public functional database, and 23137 (16.32%) co-annotated unigenes were identified in five databases (Figure 1A). We obtained the species homology distribution of *A. tatarinowii* transcripts from the Nt annotations. The matched unigenes revealed that *A. tatarinowii* had the closest homology with *Nelumbo nucifera* (12.16%) in the Nr database, followed by *Elaeis guineensis* (12.14%), *Phoenix dactylifera* (8.35%), *Macleaya cordata* (8.28%), and *Ananas comosus* (3.72%) (Figure 1B).

**Table 1:** Summary statistics of annotations for *Acorus tatarinowii* unigenes from seven publicly available databases

Database	Number annotated	Percentage (%)
Nr	77368	54.57
Nt	57501	40.56
Swissprot	58557	41.30
KEGG	62765	44.27
KOG	62928	44.39
Pfam	56187	39.63
GO	42681	30.10
Overall	84705	59.75

GO functional annotations for *A. tatarinowii* unigenes were obtained from the Nr database: 42681 unigenes (30.10%) were categorized into main GO groups of molecular function, cellular components, and biological processes. In the biological processes group, most of the unigenes were related to ‘cellular process’ (13121 unigenes), ‘biological regulation’ (5009 unigenes), ‘metabolic process’ (3173 unigenes), and ‘localization’ (3038 unigenes). In the molecular function group, 21714 unigenes were related to ‘binding,’ and 20872 unigenes were most abundant in the ‘catalytic activity’ class. In the cellular components group, most of the unigenes were involved in ‘cell’ (13439 unigenes), followed by ‘membrane part’ (11735 unigenes), and ‘organelle part’ (5289 unigenes) (Supplementary Figure S3).

In total, 62928 unigenes (44.39%) were classified into 25 unigene functional groups, with 11690 unigenes classified in the ‘general function prediction only’ group and 7981 unigenes classified in the ‘signal transduction mechanisms’ group.

### Overview of expression and MAPMAN analysis

**Figure 2:**
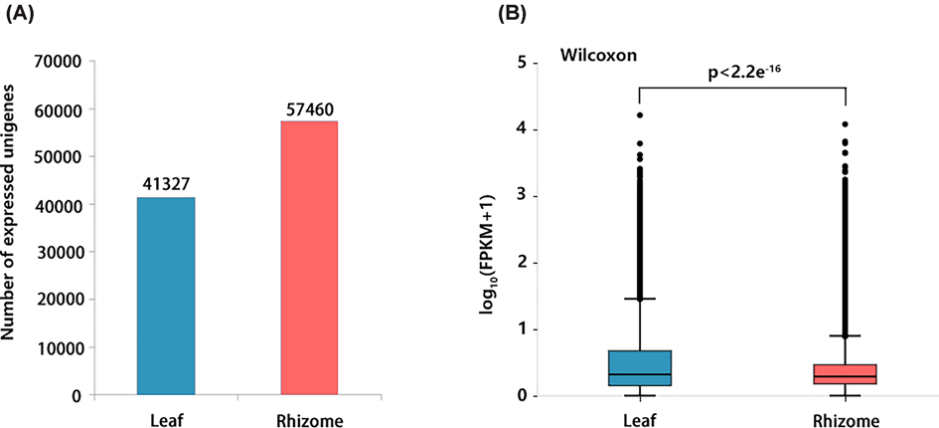
Overall expression profiles of *Acorus tatarinowii* leaf and rhizome tissues (**A**) The distribution of the number of expressed unigenes in each tissue. (**B**) Boxplot of unigenes expressed in each tissue. The x-axis represents the two tissues and y-axis represents log_10_ (FPKM + 1) values.

The expression value of all transcripts (fragments per kilobase of transcript per million mapped reads (FPKMs) > 1) in each tissue was counted. In total, 41327 and 57460 unigenes were expressed in the leaf tissue and rhizome tissue, respectively (Figure 2A). The overall gene expression level in rhizome tissue was higher than that in leaf tissue (Figure 2B).

The overall level of gene expression was analyzed in the *A. tatarinowii* by using MAPMAN software. The most common categories for the unigenes were protein (7.92%), followed by RNA (5.32%), signaling (3.03%), transport (2.46%), miscellaneous function (misc) (2.05%), and cell (1.76%) (Supplementary Figure S4). An overview of the metabolic and secondary metabolic pathways was prepared from the MAPMAN analysis based on the unigenes with FPKM > 1 (Supplementary Figure S5). In the ‘metabolic pathway’ subcategory, the highest number of unigenes was assigned to ‘secondary metabolism.’ The ‘secondary metabolic pathways’ subcategory contained 20 pathways, and most of the unigenes were in the ‘phenylpropanoids’ pathway.

### Characterization of functional genes involved in lignin biosynthesis via KEGG analysis

**Figure 3:**
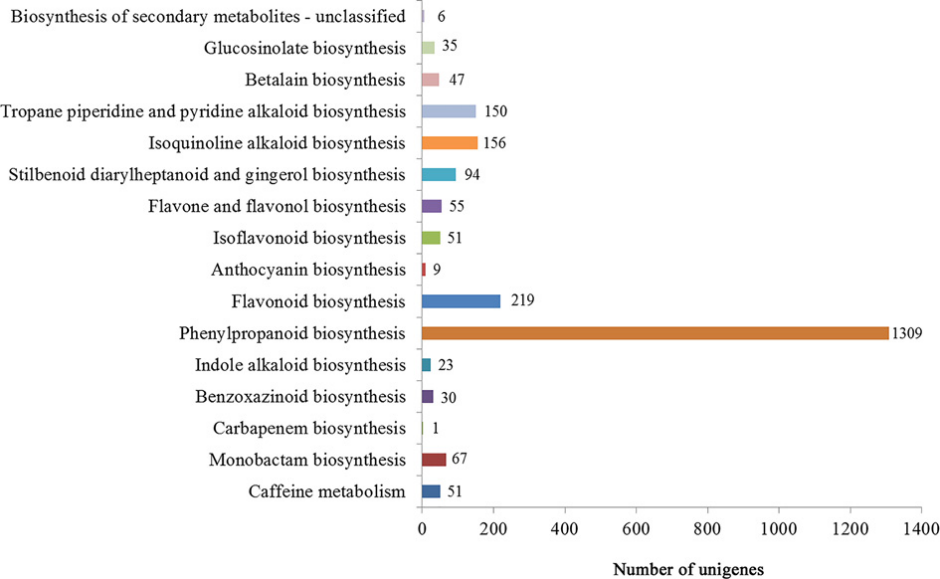
Pathway classifications for biosynthesis metabolism

**Figure 4:**
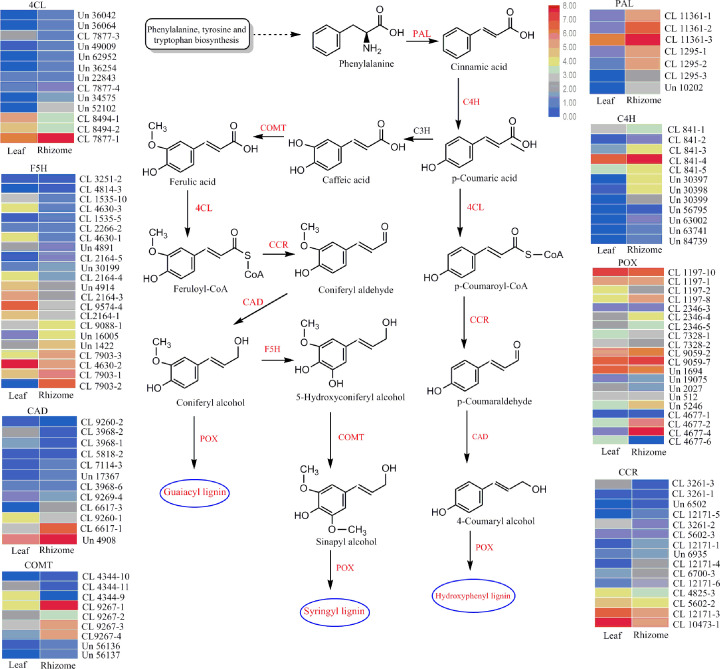
Proposed lignin biosynthetic pathways in *Acorus tatarinowii* The relative expression of the unigenes encoding enzymes from each step, presented as heatmaps. Red and blue represent high and low expression levels, respectively. Non-dashed line arrows represent the identified enzymatic reactions, and dashed line arrows represent multiple enzymatic reactions through multiple steps.

In total, 62765 unigenes (44.27%) were mapped to canonical pathways and assigned to 20 pathways based on the KEGG database. The most prevalent functional group in the KEGG pathways was ‘global and overview maps’ (13855 unigenes), followed by ‘carbohydrate metabolism’ (5653 unigenes), ‘translation’ (5232 unigenes), and ‘folding, sorting and degradation’ (4185 unigenes) (Supplementary Figure S6 and S7). The ‘biosynthesis of other secondary metabolites’ subcategory contained 16 biosynthetic pathways, including phenylpropanoid biosynthesis (ko00940), flavonoid biosynthesis (ko00941), isoquinoline alkaloid biosynthesis (ko00950), tropane, piperidine and pyridine alkaloid biosynthesis (ko00960), stilbenoid, diarylheptanoid and gingerol biosynthesis (ko00945), monobactam biosynthesis (ko00261), flavone and flavonol biosynthesis (ko00944), caffeine metabolism (ko00232), isoflavonoid biosynthesis (ko00943), betalain biosynthesis (ko00965), glucosinolate biosynthesis (ko00966), benzoxazinoid biosynthesis (ko00402), indole alkaloid biosynthesis (ko00901), anthocyanin biosynthesis (ko00942), biosynthesis of secondary metabolites-unclassified (ko00999), and carbapenem biosynthesis (ko00332) (Figure 3). Among these pathways, we annotated 1309 unigenes involved in phenylpropanoid biosynthesis and 219 unigenes involved in flavonoid biosynthesis. From further analysis of the lignin biosynthesis pathway, 107 unigenes encoding the key enzymes involved in lignin biosynthesis were identified, including PAL, C4H, COMT, 4CL, CCR, CAD, POX, F5H (Table 2). Most of the genes encoding CCR were more highly expressed in the leaf tissue, whereas the expression of most genes encoding PAL, C4H, POX, COMT, 4CL, and CAD was higher in rhizomes (Figure 4).

**Table 2:** Number of unigenes encoding the key enzymes involved in the biosynthesis of lignin metabolism in *Acorus tatarinowii*

Abbreviation	EC number	Unigene number	Enzyme name
PAL	4.3.1.24	7	Phenylalanine ammonia-lyase
C4H	1.14.14.91	13	*trans*-cinnamate 4-monooxygenase
COMT	2.1.1.68	9	Caffeic acid 3-O-methyltransferase
4CL	6.2.1.12	13	4-coumarate-CoA ligase
CCR	1.2.1.44	15	Cinnamoyl-CoA reductase
CAD	1.1.1.195	12	Cinnamyl-alcohol dehydrogenase
POX	1.11.1.7	16	Peroxidase
F5H	1.14.-.-	22	Ferulate-5-hydroxylase

### Protein–protein interaction network construction

The protein–protein interaction network between the key enzymes was predicted using STRING database (https://string-db.org/) to identify functional protein association networks. From the analysis of 107 key enzyme genes using the STRING website, seven key enzymes involved in lignin biosynthesis were annotated as node proteins: PAL, C4H, 4CL, CCR, CAD, COMT, and F5H (Supplementary Table S1). The average node degree was 9.85 and the PPI enrichment *P*-value was <1.0 × 10^−16^. This result indicated a strong association among node proteins in the PPI networks. These key enzymes may interact with each other during lignin synthesis and play crucial roles in the regulation of lignin biosynthesis (Supplementary Figure S8).

### Analysis of differentially expressed genes and specifically expressed genes in *Acorus tatarinowii*

**Figure 5:**
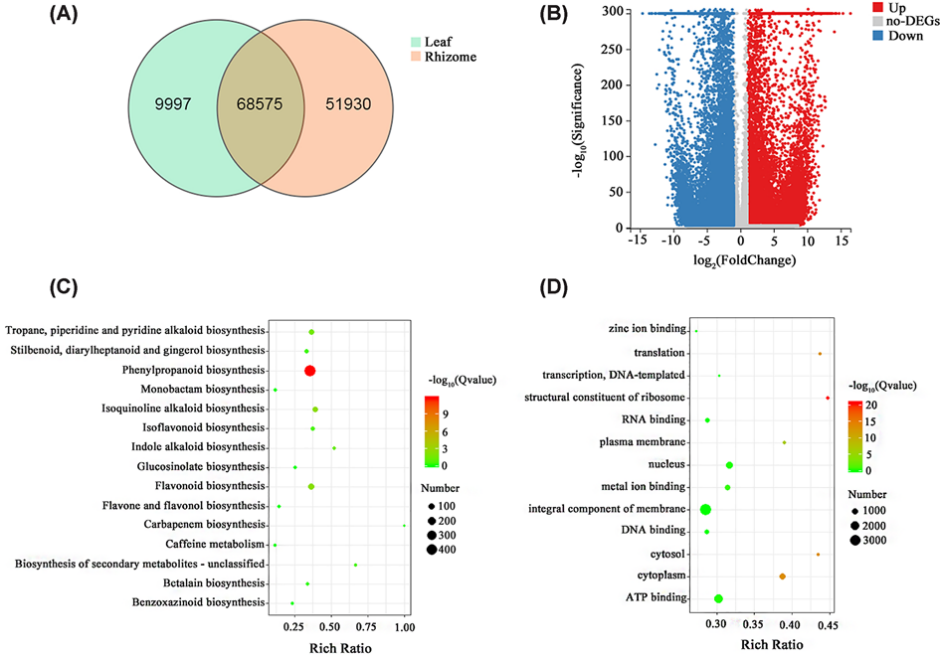
Unigenes expressed in *Acorus tatarinowii* tissues (**A**) Venn diagram of unigenes expressed in leaf and rhizome tissues. (**B**) The number of DEGs in the *A. tatarinowii* leaf and rhizome tissues. A summary of unigene numbers that were up-regulated and down-regulated between the two samples is shown. The DEGs with higher expression levels in the rhizome tissue compared with the leaf tissue were defined as up-regulated, whereas those with lower expression levels in rhizome tissue were defined as down-regulated. (**C**) KEGG classification of the up-regulated unigenes in rhizome tissue compared with leaf tissue. (**D**) GO enrichment of the up-regulated unigenes in rhizome tissue compared with leaf tissue. Abbreviation: DEG, differentially expressed gene.

In total, 68575 shared unigenes were identified in the two different tissue types of *A. tatarinowii* based on a Venn diagram analysis; 9997 and 51930 unigenes were only expressed in the leaf tissue and rhizome tissue, respectively (Figure 5A).

In the comparison of rhizome tissue with leaf tissue, 44879 unigenes were identified as significant differentially expressed genes (DEGs), which included 25602 up-regulated unigenes and 19277 down-regulated unigenes (Figure 5B). To determine the significantly enriched KEGG pathways, the 25602 up-regulated unigenes were mapped to the KEGG database. The four most common categories were ‘phenylpropanoid biosynthesis’ (288 genes), ‘flavonoid biosynthesis’ (36 genes), ‘isoquinoline alkaloid biosynthesis’ (23 genes), and ‘stilbenoid, diarylheptanoid and gingerol biosynthesis’ (21 genes). When these unigenes were mapped to the GO database, the most genes were enriched in ‘integral component of membrane’ (1952 genes), ‘ATP binding’ (973 genes), ‘nucleus’ (525 genes), and ‘DNA binding’ (344 genes) (Figure 5C, D).

The rhizome of *A. tatarinowii* is the main medicinal part of the plant. In the rhizome tissue, 51930 specifically expressed unigenes were identified; the significantly enriched KEGG pathways were ‘phenylpropanoid biosynthesis’ (468 unigenes), ‘flavonoid biosynthesis’ (80 unigenes), ‘isoquinoline alkaloid biosynthesis’ (61 unigenes), and ‘tropane, piperidine and pyridine alkaloid biosynthesis’ (55 unigenes). The significantly enriched GO terms were ‘integral component of membrane’ (3329 unigenes), ‘ATP binding’ (1958 unigenes), ‘nucleus’ (1226 unigenes), and ‘cytoplasm’ (904 unigenes) (Supplementary Figure S9 and S10).

### Identification of transcriptome factors involved in lignin biosynthesis in *Acorus tatarinowii*

**Figure 6:**
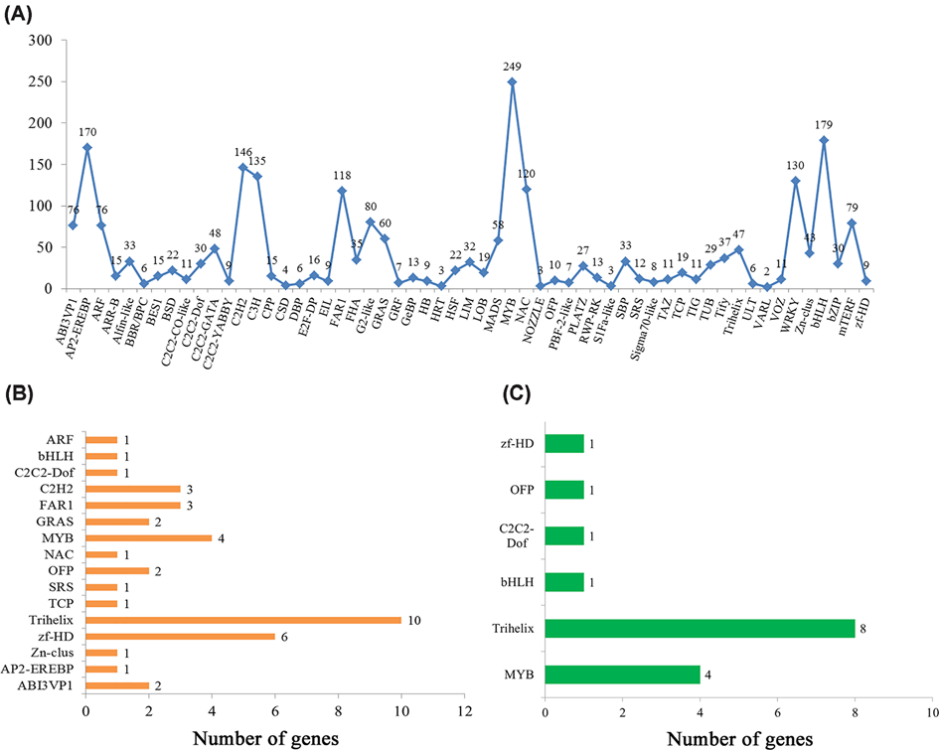
Analysis of transcription factors (**A**) Classification of transcription factor families. (**B**) Classification of transcription factors involved in the biosynthesis of other secondary metabolites. (**C**) Classification of transcription factors involved in lignin biosynthesis.

In the *A. tatarinowii* transcriptome database, 2426 unigenes were mapped to 57 transcriptome factor (TF) families. Mostly, unigenes in the MYB family were enriched (249 unigenes), followed by bHLH (179 unigenes), AP2-EREBP (170 unigenes), C2H2 (146 unigenes), C3H (135 unigenes), WRKY (130 unigenes), NAC (120 unigenes), and FAR1 (118 unigenes) (Figure 6A). From the KEGG pathway analysis of all TFs, 40 TFs related to the biosynthesis of other secondary metabolites were identified. Among these, 10 unigenes were enriched in the Trihelix family, and in the MYB (6 unigenes), zf-HD (6 unigenes), FAR1 (3 unigenes), C2H2 (3 unigenes), and GRAS (3 unigenes) families (Figure 6B). Sixteen unigenes involved in lignin biosynthesis were identified, including Trihelix (8 unigenes), MYB (4 unigenes), C2C2-Dof (1 unigene), OFP (1 unigene), zf-HD (1 unigene), and bHLH (1 unigene) (Figure 6C), and these TFs related to lignin biosynthesis regulated the same key enzyme (POX) (Supplementary Table S2).

### Structural characteristics of peroxidase involved in lignin biosynthesis

**Figure 7:**
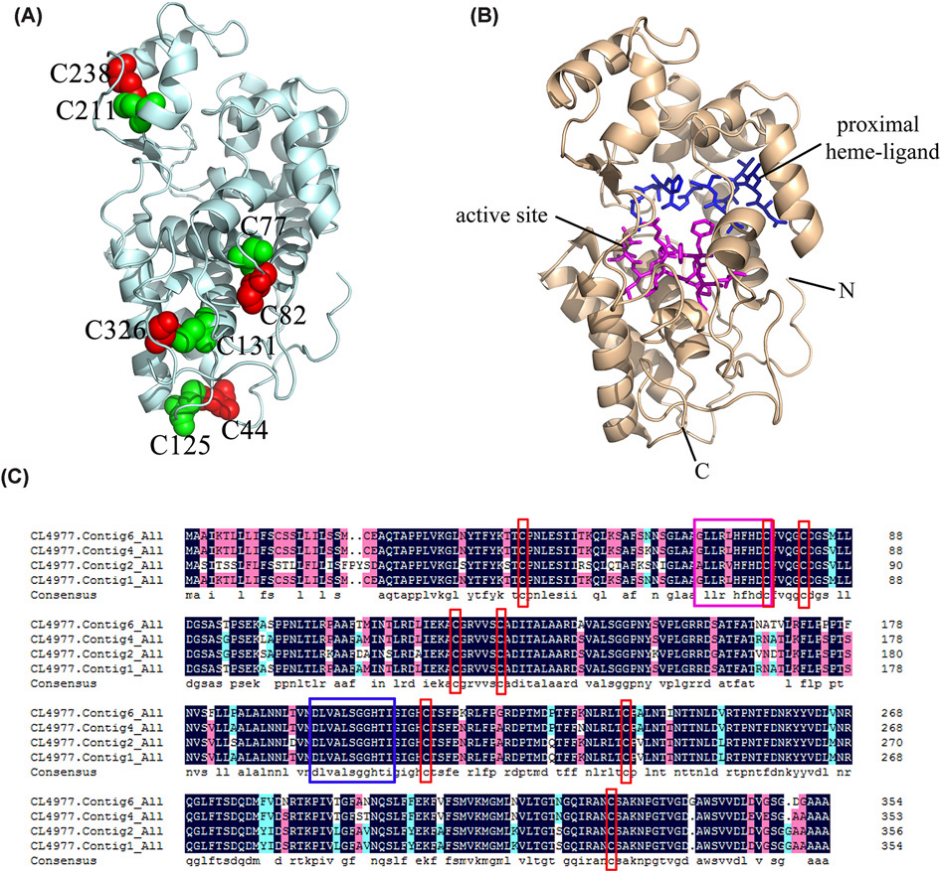
Spatial structure model of peroxidase (**A**) Four disulfide bonds (C211–C238, C77–C82, C131–C326, and C125–C44) are shown as spheres and colored in red or green. (**B**) The active site and proximal heme-ligand of the peroxidases are shown in pink and blue, respectively). (**C**) Alignment of four amino acid sequences of peroxidases in *A. tatarinowii*. Black and pink show identical and similar amino acids, respectively. The red box highlights four conserved disulfide bonds, the blue box highlights the proximal heme-ligand signature, and the pink box highlights the active site.

The alignment of the four peroxidase amino acid sequences revealed that they shared a very high sequence identity (92.67%, Figure 7C). A 3D structural model of peroxidase (CL4977.Contig4, Range P29-N330, Coverage 0.85) was constructed based on the crystal structure of peroxidase from *Sorghum bicolor* (PDB ID: 5aog, 64.57% sequence identity) by using SWISS-MODEL (https://swissmodel.expasy.org/) and PyMOL software. The structure of peroxidase (CL4977.Contig4) is presented in Figure 7. The predicted tertiary structure indicated that the peroxidase contained two different domains, comprising 11 α-helixes. ‘AAGLLRLHFHDC’ (A66–C77) is the peroxidase active site signature (colored in pink, Figure 7B), and ‘DLVALSGGHTI’ (D196–I206) is the peroxidase proximal heme-ligand signature (colored in blue, Figure 7B). Moreover, the peroxidases contained four conserved disulfide bonds (C211–C238, C77–C82, C131–C326, and C125–C44, Figure 7A).

### qRT-PCR validation of gene expression of key enzymes

**Figure 8:**
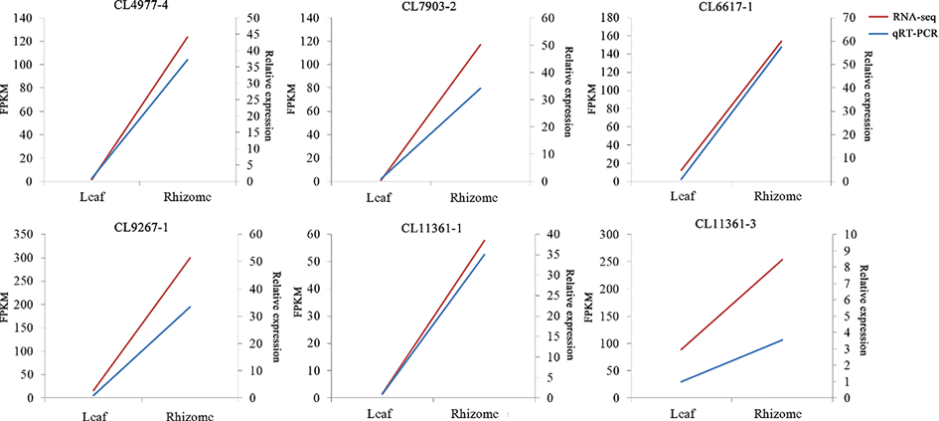
qRT-PCR and FPKM-value analysis of six genes encoding enzymes involved in lignin biosynthesis in *Acorus tatarinowii* The expression of POX (CL4977-4), F5H (CL7903-2), CAD (CL6617-1), COMT (CL9267-1), and PALs (CL11361-1 and CL11361-3) was determined relative to the expression of the actin gene (Unigene12762); three technical replicates of each sample were analyzed.

The gene expression levels of POX (CL4977-4), F5H (CL7903-2), CAD (CL6617-1), COMT (CL9267-1), and PAL (CL11361-1 and CL11361-3) were evaluated in the rhizome and leaf tissues of *A. tatarinowii* by using qRT-PCR. The relative expression of these genes was significantly higher in rhizome tissue than in leaf tissue, which was consistent with the transcriptome data (Figure 8).

## Discussion

*A. tatarinowii* is a well-known traditional medicinal plant [21]: its sedative, digestive, analgesic, diuretic, and antifungal effects are recorded in the Chinese Pharmacopoeia [22]. Even though lignins are considered to be the major bioactive components of *A. tatarinowii*, there are comparatively few studies relating to lignins [23]. To identify the unigenes involved in the lignin biosynthesis pathway, we constructed a large number of gene sequences to help identify more key enzymes to assist research of lignin biosynthesis in *A. tatarinowii*. Based on the *A. tatarinowii* transcriptome database, 141777 unigenes were uncovered, with an average length of 1108 nt and an N50 length of 2062 nt; 51352 unigenes (36.22%) were longer than 1000 nt. Furthermore, the relative gene expression of POX, F5H, CAD, COMT, and PAL was examined using qRT-PCR, and found to be consistent with our transcriptional data. These results confirmed the high quality of the transcriptome assembly and the accuracy of our *A. tatarinowii* transcriptome database. The availability of *A. tatarinowii* transcriptome data will support the study of biological metabolism and functional genes of other homologous species.

When the DEGs were mapped to KEGG pathways, the expression level of 25602 unigenes in rhizome tissues was significantly up-regulated, these were mainly associated with the pathways of ‘phenylpropanoid biosynthesis,’ ‘flavonoid biosynthesis,’ and ‘isoquinoline alkaloid biosynthesis.’ A total of 51930 unigenes were identified as specifically expressed in rhizome tissue, and were mainly associated with the following KEGG pathways: ‘phenylpropanoid biosynthesis,’ ‘flavonoid biosynthesis,’ and ‘isoquinoline alkaloid biosynthesis. These data may confirm the medicinal value of *A. tatarinowii* from rhizome tissues at the molecular level. KEGG annotations revealed eight key enzymes related to lignin biosynthesis, namely PAL, C4H, COMT, 4CL, CCR, CAD, POX, and F5H. These key enzymes play important roles in the molecular mechanisms of lignin synthesis in *A. tatarinowii*. Compared with the leaf tissue, unigenes encoding the PAL, C4H, COMT, CAD, and POX enzymes were more highly expressed in rhizome tissue. COMT, CAD, and POX have been reported to play important roles in the process of lignin biosynthesis, and revealed that the expression levels of key enzymes genes may regulate the synthesis of lignin [24–26].

In the transcriptome data of *A. tatarinowii*, 2426 unigenes were successfully annotated to 57 transcription factor families. Mostly, unigenes in the MYB family were enriched. Studies have reported that MYB transcription factors are involved in lignin biosynthesis in many plant species, including *Arabidopsis, Loquat undergoes*, and *poplar*, and play a role in the formation of secondary cell walls [27–29]. Through KEGG pathway analysis of TFs, 16 TFs involved in lignin biosynthesis in *A. tatarinowii* were identified, and these TFs regulated the same key enzyme (peroxidase). These results indicate that peroxidase may play a key role in the regulation of lignin biosynthesis in *A. tatarinowii*. Hence, the TFs regulating peroxidase are important candidates for future studies of the regulation of lignin biosynthesis in *A. tatarinowii.* However, RNA-Seq is not appropriate for the detection of regulatory genes with low expression levels [30,31]. For this reason, many TFs are hard to detect, which has obstructed research into their functions; however, they are still very important, because a small increase in the expression of TFs may have a dramatic effect.

The four sequences of peroxidase in *A. tatarinowii* had well-conserved amino acid sequences, spatial structures, active sites, and proximal heme-ligand signatures. Moreover, the peroxidase had eight conserved cysteine residues, which serve as the site of oxidation by peroxides. Other research has shown that the peroxidases active site (AAGLLRLHFHDC) and proximal heme-ligand (DLVALSGGHTI) played a catalytic role by forming covalent bonds with Ca^2+^, Na^+^, and Fe^2+^; of these, Ca^2+^ and Na^+^ are the proximal and distal conserved binding sites. The incorporation of metal ions plays a regulatory role in the catalytic process of peroxidase [32,33].

Lignin is a promising material for pharmaceutical and biomedical applications owing to its biocompatibility, low cytotoxicity, antioxidant and antimicrobial properties. In medicine, it can be used as an auxiliary material to improve the oral bioavailability of the drug [34]. In addition, lignin can increase the stability of the antimicrobial properties of wound dressings [35,36]. Lignin-based hydrogels, which combine lignin with other polymers, have been used for gene delivery [37], drug delivery [38], and tissue engineering [39,40].

This is the first transcriptome sequencing study of two tissues of *A. tatarinowii* using RNA-Seq technology. The obtained data have provided comprehensive information on the gene sequences and expression level of genes in *A. tatarinowii*, and will support more detailed studies of the molecular mechanism of lignin biosynthesis.

## Materials and methods

### Plant material and RNA extraction

Three plants of *A. tatarinowii*, which were cultivated in the natural environment of the pharmaceutical garden of Anhui University of Chinese Medicine, were harvested in April 2019. Fresh plants were quickly cleaned with sterile water and wiped clean with filter paper. The tissues (leaf and rhizome) were separately collected into 50-mL centrifuge tubes, frozen quickly in liquid nitrogen for 6 h, and then stored at −80 °C for RNA extraction. An independent experiment was conducted for each *A. tatarinowii* tissue, with three biological replicates used for each treatment.

### cDNA library construction and sequencing (RNA-Seq)

Each plant tissue sample of RNA was extracted by using the RNA Plant Kit (Aidlab Biotech, Beijing, China) in accordance with the manufacturer’s protocol. The mRNA from each sample was isolated from the total RNA by using beads with oligo(dT), and lysed into short fragments using fragmentation buffer. Random N6 primers were used to reverse-transcribe the short fragments of mRNA obtained; the first-strand cDNA was synthesized as a template, and two-strand cDNA was synthesized to form double-stranded cDNA by using dNTPs, RNase H, and DNA polymerase I. The obtained short cDNA fragment was ligated to the adapter, and the entire fragment was selected for PCR amplification. Finally, the quantification of cDNA library per tissue was detected, and cDNA library of plant tissues was constructed by BGISEQ-500 [41].

### Transcriptome *de novo* assembly and unigene functional annotation

To obtain high-quality clean data, raw reads were filtered to remove low-quality reads with greater than 20% Q-value < 20 bases, ambiguous reads containing >5% unknown base (N), and for non-coding RNA by SOAPnuke and Trimmomatic software. Full-length transcripts were *de novo* assembled through a broad range of expression levels and sequencing depths by using Trinity software (v2.0.6), default parameters (K-mer = 25, group pairs distance = 400) [42]. These contigs were then further processed with Trinity to remove the redundant transcripts by using TGICL (v2.0.6) software, and finally, to generate unigenes.

Unigenes were mapped to several public databases using BLAST (version 2.2.23, e-value ≤ 1 × 10^−5^), including Nt (ftp://ftp.ncbi.nlm.nih.gov/blast/db), Nr (ftp://ftp.ncbi.nlm.nih.gov/blast/db), COG (https://www.ncbi.nlm.nih.gov/COG/), KEGG (http://www.genome.jp/kegg), and Pfam (http://pfam.xfam.org). According to the Nr annotation, the GO (http://geneontology.org) annotation of unigenes was obtained using the Blast2GO program (https://www.blast2go.com) [43].

### MAPMAN annotation and protein–protein interaction network construction

MAPMAN (http://mapman.gabipd.org/) is a tool for plant metabolism distribution research that performs large gene data enriched to diagrams of plant metabolic pathways [44]. All *A. tatarinowii* gene sequences were annotated by the MAPMAN database (http://www.plabipd.de/portal/mercator-sequence-annotation) to form independent species mapping. The expression level of unigenes in *A. tatarinowii* rhizome tissue was analyzed using MAPMAN software.

Based on *Arabidopsis thaliana* genomic data, the STRING online tool was used to analyze the genes encoding the key enzyme involved in the biosynthesis of lignin metabolism, and to predict the interactions between them. The protein–protein interactions of key enzymes were selected with score (median confidence) >0.4, and the node proteins were selected based on their association with other proteins. In a PPI network, an interactive network with a higher degree of association with other interactive networks plays a more important role [45].

### Analysis of DEGs

RNA-seq has emerged as a powerful technology to measure gene expression and tissue specificity at the whole-transcriptome level. In the present study, the transcriptome data were evaluated to determine the differences in gene expression in the leaf and rhizome of *A. tatarinowii*. We calculated FPKM values to quantify the expression levels of each unigene. From the analysis of the DEGs, high-quality reads were assembled on the final transcriptome using Bowtie2 (version 2.2.5). Differential gene expression in two tissues was determined using the PossionDis method, which was performed as described: FC (fold change) ≥ 2.00 and FDR ≤ 0.001 [46]. Moreover, functional enrichment of the DEGs was annotated by the GO functional database by using GO-Term Finder software. KEGG pathway annotation was applied to significant DEGs.

### Identification of TFs

In order to identify the transcription factor family represented in the *A. tatarinowii* transcriptome, Getorf (http://emboss.sourceforge.net/apps/cvs/emboss/apps/getorf.html) was applied to detect the open reading frames (ORFs) of the unigenes, and then to align them with the protein domains of transcription factors. In our transcriptome, unigene-encoded protein sequences were probed against and matched with all the transcription factor protein sequences in the Plant transcription factor database (PlantTFDB). Finally, the transcription factors were successfully predicted in the *A. tatarinowii* transcriptome [47].

### Analysis of the structural characteristics of peroxidase

Four amino acid sequences of Pox were submitted to the SmartBlast in NCBI (http://www.ncbi.nlm.nih.gov/) to search for enzyme sequences of homologous species. The alignment of four ORFs of POX (peroxidase) was compared using DNAMAN software. SWISS-MODEL (https://swissmodel.expasy.org/) and PyMOL were applied to analyze the 3D structure model of the peroxidase protein [48].

### qRT-PCR validation

The key enzyme genes involved in lignin synthesis were determined by quantitative real-time PCR in accordance with the instructions of Power SYBR® Green PCR Master Mix (Roche) and Quantstudio multiplex real-time PCR instrument (Life Technologies, U.S.A.). Based on transcriptome sequences, special PCR primers were designed for different genes using Primer Premier 6.0 and Beacon designer 7.8 software (Supplementary Table S3). We used 20 µl of reaction mixture, including 1 µL of diluted cDNA, 10 µl of Power SYBR® Green Master Forward Primer, 8 µL of SDW, 0.5 µL of forward primer (10 µM), and 0.5 µL of reverse primer (10 µM). The real-time PCR program used was 95 °C for 1 min, 40 cycles of 95 °C for 15 s, and 63 °C for 25 s. For each sample, three technical replicates of the qRT-PCR analyses were repeated, and the mean of the three replicates was used as the PCR result. The actin gene (Unigene12762) served as the internal control. The relative expression levels of the selected genes were calculated using the 2^(−ΔΔ*C*_t_)^ method [49].

## Supplementary Material

Supplementary Figures S1-S10 and Tables S1-S3

## Data Availability

The RNA-seq datasets from two *A. tatarinowii* tissues were deposited in the NCBI Sequence Read Archive (SRA) database (accession: SRP213064).

## Abbreviations

CAD: cinnamyl-alcohol dehydrogenase
CCR: cinnamyl-CoA reductase
COMT: caffeic acid 3-O-methyltransferase
C4H: cinnamate 4-hydroxylase
DEG: differentially expressed gene
FDR: False Discovery Rate
FPKM: fragments per kilobase of transcript per million mapped read
F5H: ferulate-5-hydroxylase
ORF: open reading frame
PAL: phenylalanine ammonia-lyase
POX: peroxidase
PPI: protein–protein interaction
qRT-PCR: real-time quantitative Polymerase Chain Reaction
SDW: sterile distilled deionized water
TF: transcriptome factor
4CL: 4-coumaroyl CoA-ligase
