# Supplementary material for: Exploring the biosynthetic pathway of lignin in Acorus tatarinowii Schott using de novo leaf and rhizome transcriptome analysis
Source: Biosci Rep. 2021 Jun 11;41(6):BSR20210006. doi: 10.1042/BSR20210006 (PMC8200657; doi:10.1042/BSR20210006)
Supplement: Supplementary Figures S1-S10 and Tables S1-S3 [file BSR-2021-0006_supp.pdf]

Exploring the biosynthetic pathway of lignin in *Acorus tatarinowii* Schott using *de novo* leaf and rhizome transcriptome analysis

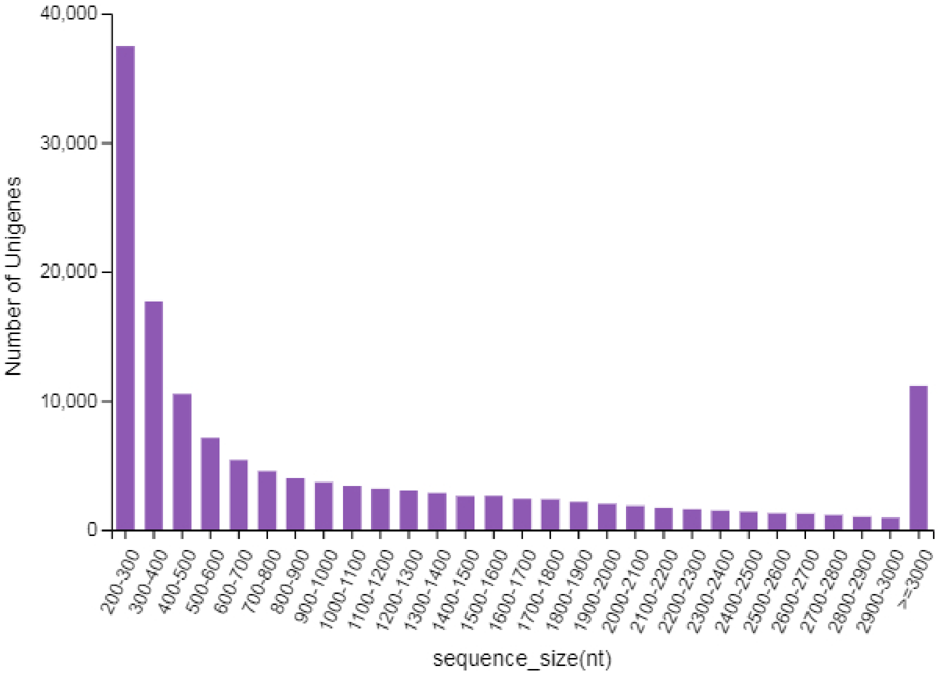

Supplementary Figure S1 Length distribution of the assembled transcript sequences of *Acorus tatarinowii* Schott **.**

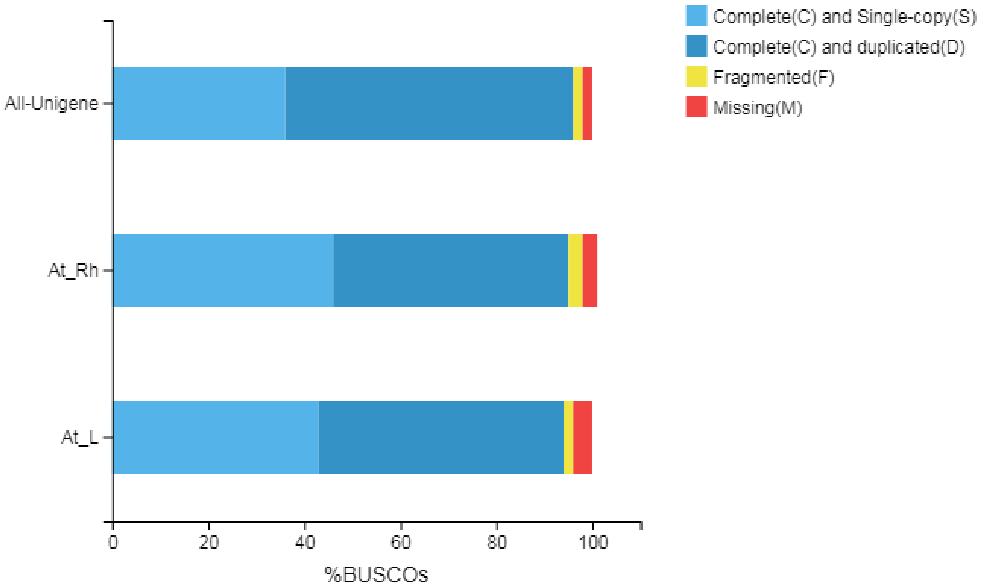

Supplementary Figure S2 Match the sequence of the BUSCO database; F (fragmented): only part of the sequence can be compared to the BUSCO database; D (duplicate): multiple genes are aligned with the same BUSCO; M (missing) is filtered out of the sequence.

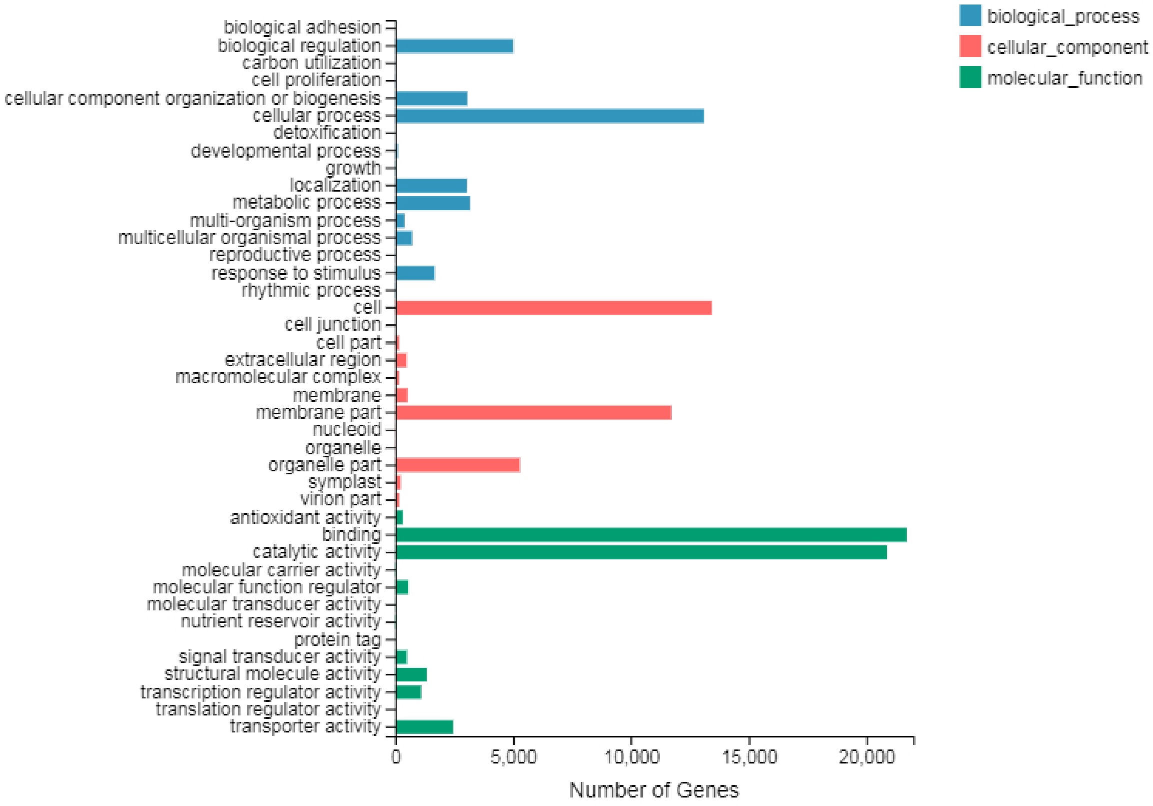

Supplementary Figure S3 GO function classifcations of *Acorus tatarinowii* Schott transcriptome.

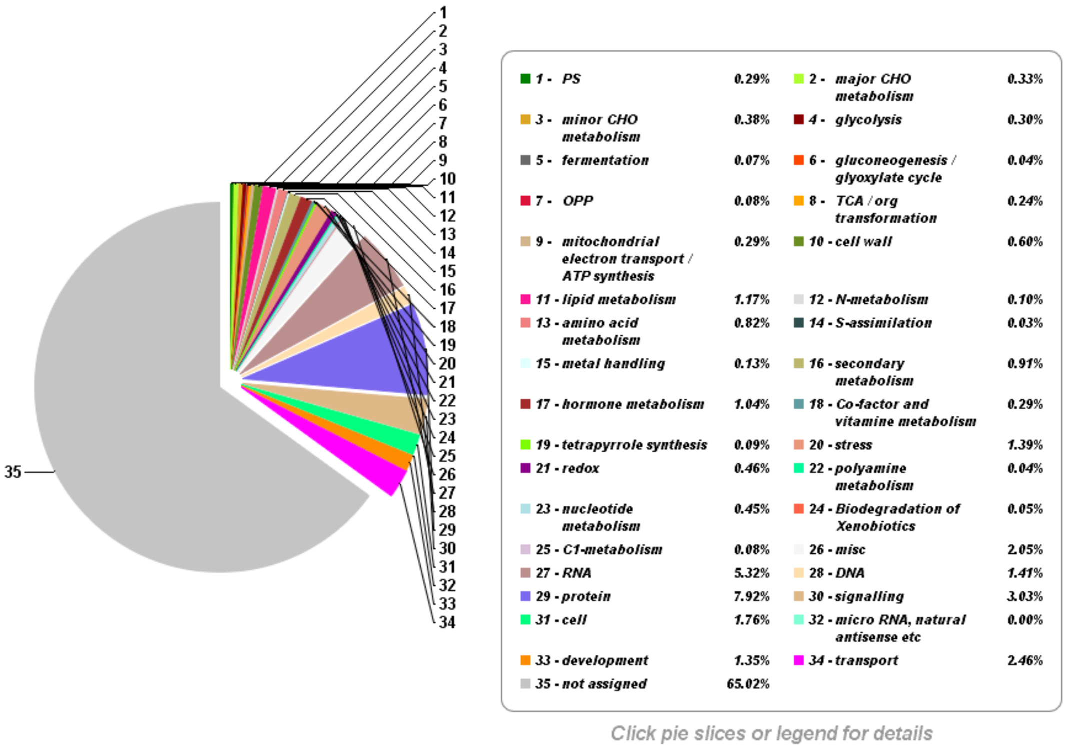

Supplementary Figure S4 Annotation of unigenes in *Acorus tatarinowii* Schott using MAPMAN software.

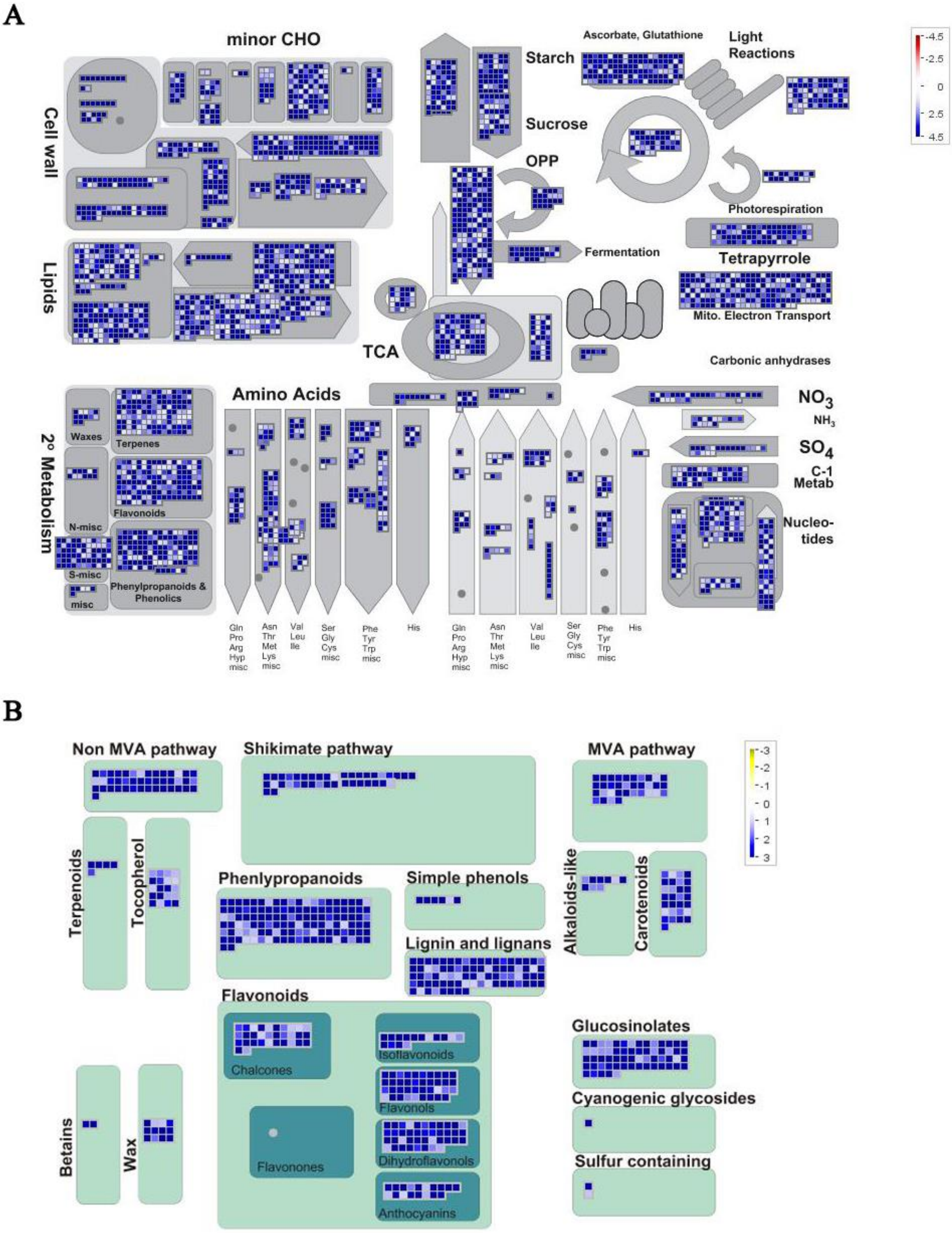

Supplementary Figure S5 Overview of metabolism and secondary metabolic pathways in *Acorus tatarinowii* Schott using MAPMAN analysis. (A) overview of metabolism (B) secondary metabolic pathways

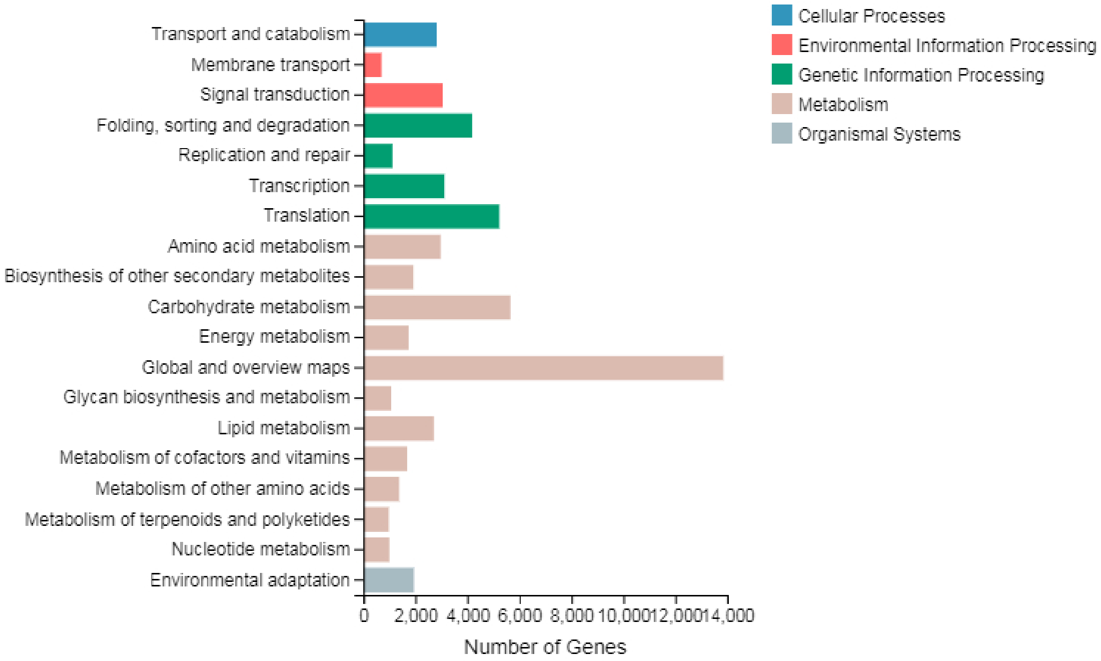

Supplementary Figure S6 KEGG functional annotated in *Acorus tatarinowii* Schott.

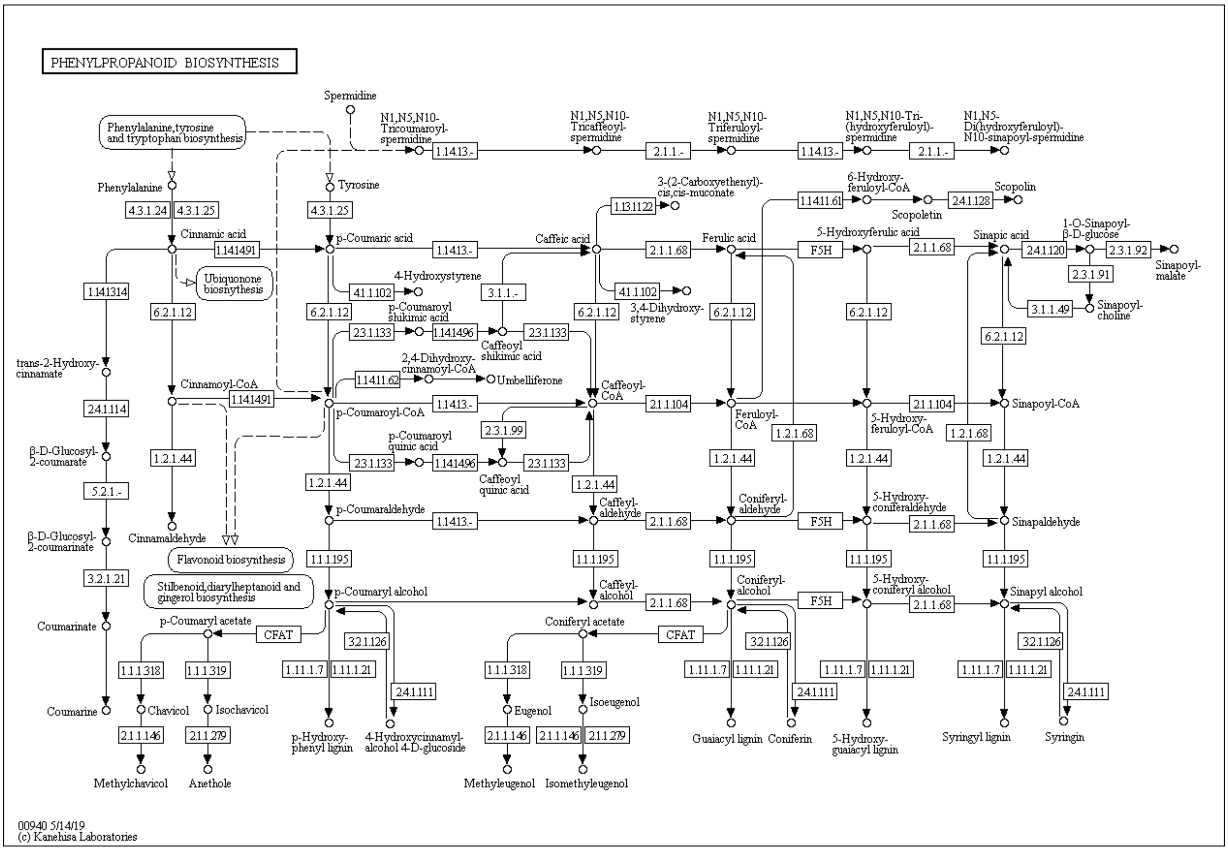

Supplementary Figure S7 Phenylpropanoid biosynthesis in *Acorus tatarinowii* Schott based on the KEGG database.

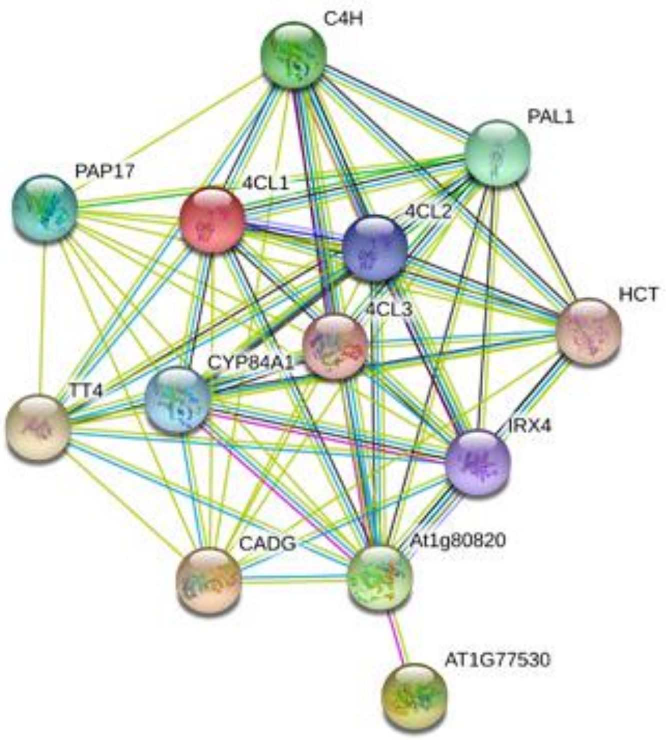

Supplementary Figure S8. Protein–protein interaction network diagram of the node proteins.

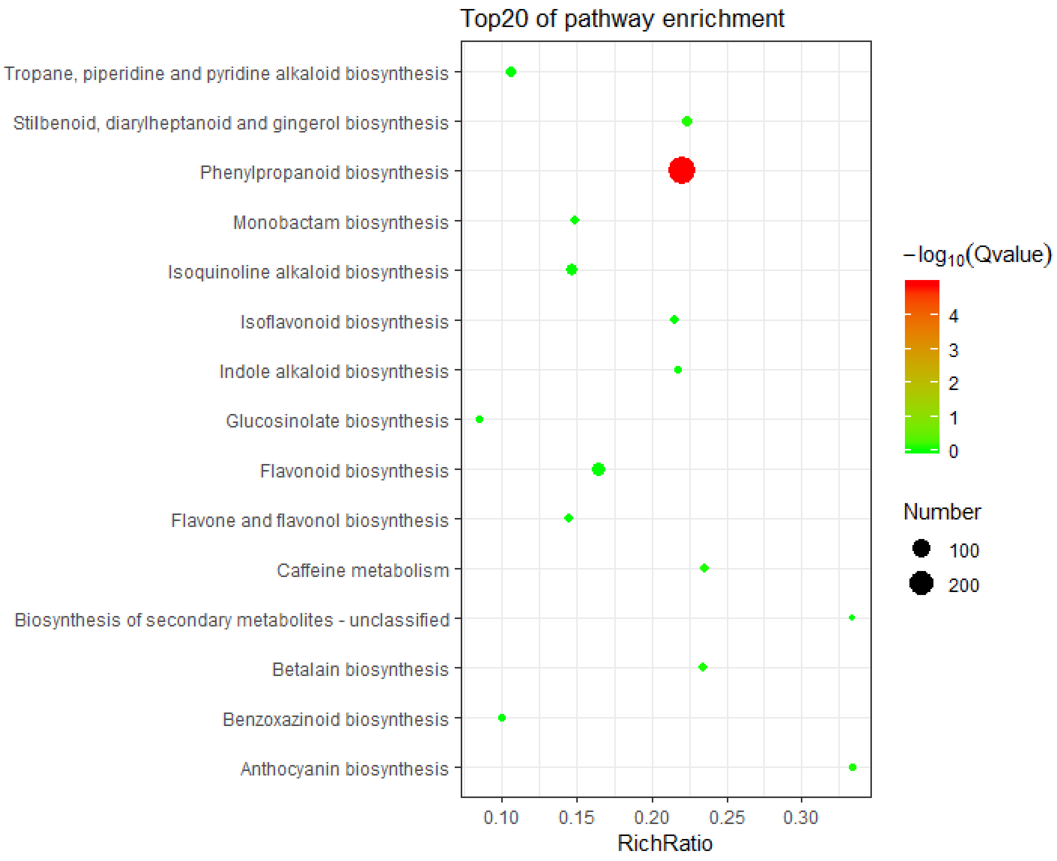

Supplementary Figure S9 KEGG classification of specific expression up-regulated unigenes in *Acorus tatarinowii* Schott rhizome tissue.

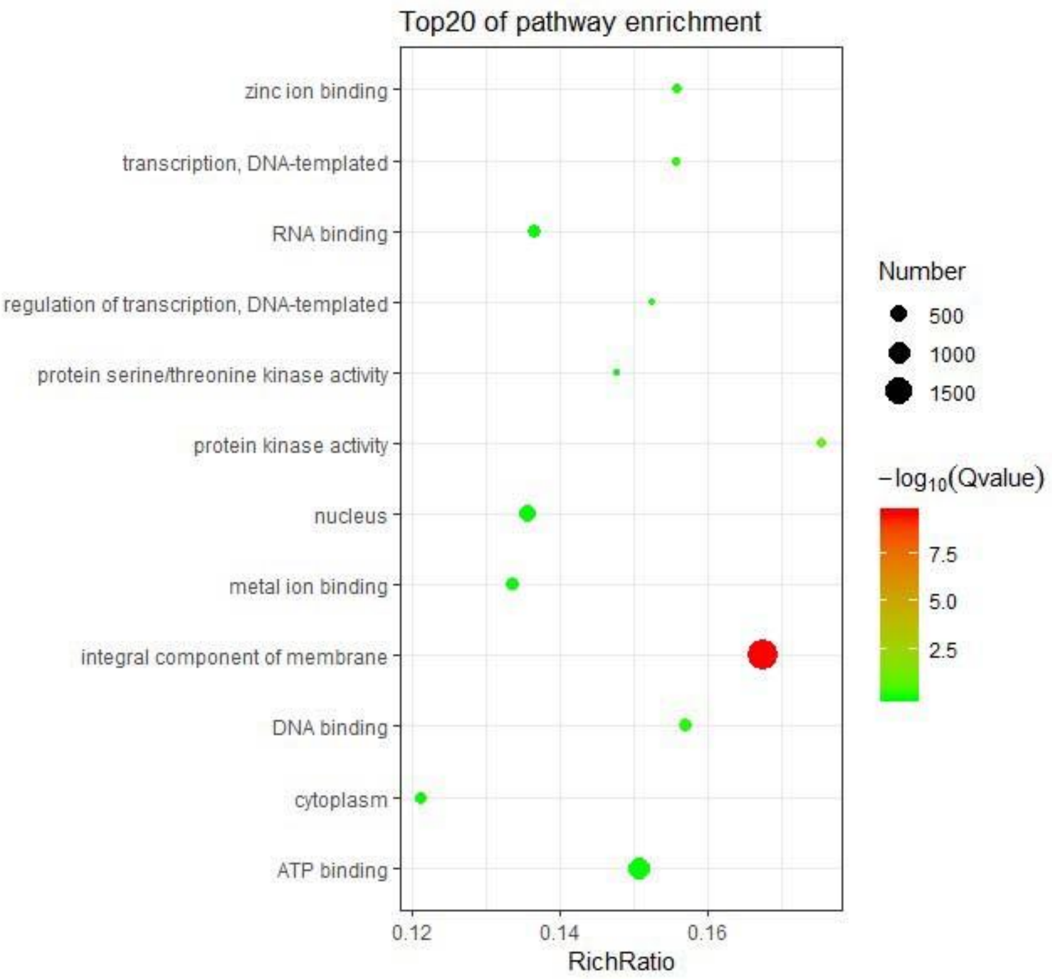

Supplementary Figure S10 GO classification of specifc expression up-regulated unigenes in *Acorus tatarinowii* Schott rhizome tissue.

Supplementary Table S1 Annotation of the 13 key node proteins.

Node protein	Node accession	Node protein annotation
PAL	AT2G37040.1	phenylalanine ammonia-lyase1 (PAL1)
C4H	AT2G30490.1	trans-cinnamate 4-monooxygenase (C4H)
F5H	AT4G36220.1	Ferulic acid 5-hydroxylase 1(CYP84A1)
4CL	AT1G51680.1	4-coumarate-CoA ligase1 (4CL1)
4CL	AT3G21240.1	4-coumarate-CoA ligase 2 (4CL2)
4CL	AT1G65060.1	4-coumarate-CoA ligase 3 (4CL3)
CCR	AT1G80820.1	cinnamoyl-CoA reductase 2 (AT1G80820)
CAD	AT1G72680.1	probable cinnamyl alcohol dehydrogenase 1 (CADG)
PAP17	AT3G17790.1	Purple acid phosphatase 17 (PAP17)
TT4	AT5G13930.1	chalcone and stilbene synthase family protein (TT4)
HCT	AT5G48930.1	quinate hydroxycinnamoyltransferase (HCT)
COMT	AT1G77530	putative caffeic acid 3-O-methyltransferase (AT1G77530)
CCR	AT1G15950.1	cinnamoyl-CoA reductase 1 (IRX4)

Supplementary Table S2 The detail of TFs involed in lignin biosynthesis.

Gene ID	At_L FPKM	At_Rh FPKM	TFs	Enzyme
Unigene512_All	4.46	4.62	zf-HD	peroxidase
CL2346.Contig3_All	1.32	1.36	Trihelix	peroxidase
CL2346.Contig4_All	3.75	11.65	Trihelix	peroxidase
CL2346.Contig5_All	3.38	9	Trihelix	peroxidase
CL7328.Contig1_All	6.65	5.66	Trihelix	peroxidase
CL7328.Contig2_All	4.52	3.19	Trihelix	peroxidase
CL9059.Contig2_All	34.39	49.4	Trihelix	peroxidase
CL9059.Contig7_All	68.56	95.23	Trihelix	peroxidase
Unigene5246_All	6.85	15.12	Trihelix	peroxidase
Unigene2027_All	2.56	1.69	OFP	peroxidase
CL1197.Contig10_All	101.83	74.89	MYB	peroxidase
CL1197.Contig1_All	28.04	23.77	MYB	peroxidase
CL1197.Contig2_All	10.24	3.2	MYB	peroxidase
CL1197.Contig8_All	10.78	28.32	MYB	peroxidase
Unigene19075_All	1.31	1.83	C2C2-Dof	peroxidase
Unigene1694_All	62.9	45.09	bHLH	peroxidase

Supplementary Table S3 Quantitative Real-Time PCR (qRT-PCR) primers and conditions.

Genbank Accession	Primer Sequences (5' to 3')	Annealing (°C)
Unigene12762	TGACCTTGAAGTACCCCATTGA<br>GAAGCATAGAGGGAAAGCACAG	58
CL4977-4	TCCTGATCTTCTCCTGCTCTTC<br>TTTACTGAAGGCGGATTTGAGC	60
CL7903-2	TGAAGCTCAGCCGTGACAACATC<br>TCCGTCATCGCCCACTCTATCG	62
CL6617-1	AATCCCAAACGGCTATCCATTG<br>AAACTTGACAGCCATATGACCG	58
CL9267-1	CTTGCCTCTACTCCATGATCCT<br>ACGTCAGTATTTTGTAGCTCGC	60
CL11361-1	GAACACTCTCATGCAGGGCTA<br>CCGTTAAGAGACCCGCTATGTA	59
CL11361-3	GAGCTCATCCGGTTCTTGAAC<br>CTTCCAGAAGCTCCCAACGAAT	59
